# Characterising the diagnosis of genetic maculopathies in a real-world private tertiary retinal practice in Australia: protocol for a retrospective clinical audit

**DOI:** 10.1080/07853890.2023.2250538

**Published:** 2023-08-26

**Authors:** Alexis Ceecee Britten-Jones, Demi Markakis, Robyn H. Guymer, Ming-Lee Lin, Simon Skalicky, Lauren N. Ayton, Heather G. Mack

**Affiliations:** a Ophthalmology, Department of Surgery, University of Melbourne, Melbourne, Australia; b Centre for Eye Research Australia, Royal Victorian Eye and Ear Hospital, Melbourne, Australia; c Department of Optometry and Vision Sciences, University of Melbourne, Melbourne, Australia; d Cabrini Hospital, Malvern, Australia; e Faculty of Medicine, Nursing and Health Sciences, Monash University, Clayton, Australia; f Eye Surgery Associates, East Melbourne, Australia

**Keywords:** Age-related macular degeneration, inherited retinal disease, inherited macular disease, macular dystrophy, fundus autofluorescence, retrospective clinical review

## Abstract

**Purpose:**

Accurate diagnosis of macular atrophy is paramount to enable appropriate treatment when novel treatments for geographic atrophy and macular dystrophies become available. Genetic testing is useful in distinguishing between the two conditions but is not feasible for the majority of patients in real-world clinical practice. Therefore, we aimed to investigate the potential misdiagnosis of inherited macular dystrophy as age-related macular degeneration (AMD) in real-world ophthalmic practice to assist in the development of guidelines to improve diagnostic accuracy while minimizing genetic testing for targeted patients.

**Methods:**

Retrospective review of the medical records of patients diagnosed with AMD, which included imaging, between 1995 and 2023 from a large multidisciplinary private ophthalmic practice in Australia. We will use a stepwise method to screen for probable cases of macular dystrophy, followed by a consensus review by an expert panel. The outcomes are (1) to determine the potential misdiagnosis rate of macular dystrophy as atrophic AMD by retinal specialists and general ophthalmologists; (2) to identify clinical imaging modalities that are most useful for differentiating macular dystrophy from atrophic AMD; and (3) to establish preliminary guidance for clinicians to improve the diagnosis of macular atrophy from AMD in practice, and thereby target cost-efficient genetic testing.

**Discussion:**

Improving the diagnostic accuracy of both AMD and macular dystrophy, while ensuring cost-efficient genetic testing, will improve the targeted treatment of macular diseases when emerging treatments become available.

## Introduction

The macula occupies about 10% of the area of the retina, temporal to the optic disc, and is responsible for central vision. The retina is divided into the inner and outer segments; the inner retina contains cell bodies and synapses of retinal ganglion, bipolar, horizontal, and amacrine cells, and the outer retina include photoreceptors cells and retinal pigment epithelium (RPE), through to the choroid. Macular diseases are associated with reduced visual acuity, reduced colour vision and central scotomata (blind spots in the visual field).

Age-related macular degeneration (AMD) is a disease of the outer retina in the macula location, and is the leading cause of irreversible vision loss in people aged ≥50 years in developed nations [1]. In Australia, the prevalence of early AMD has been reported to be 14.8% in non-indigenous Australians over the age of 50 [2], with the burden of AMD projected to rise among our ageing population [3]. AMD is a complex multi-genic and multifactorial disorder, for which there are no commercially available genetic confirmatory tests. Late-stage complications of AMD include neovascular AMD (nAMD) and geographic atrophy (GA), both of which have vision-threatening complications [4]. GA is characterized by loss of photoreceptors and RPE in the outer retina and presents with a sharply defined border and exposed underlying choroidal blood vessels [5].

Anti-vascular endothelial growth factor drugs have revolutionized the treatment of neovascular complications associated with neovascular AMD (nAMD), leading to reduced rates of vision loss, and in some cases, vision improvement [6]. Recently, pegcetacoplan, a complement 3 inhibitor, was approved by the United States Federal Drug Administration for the treatment of GA [7], and advanced-stage clinical trials of other complement-based therapeutics are underway [8–10].

Inherited retinal diseases (IRDs) are a group of heterogeneous, degenerative retinal conditions that represent the most common cause of legal blindness in adults of working age in Australia [11] and the UK [12]. The macula-only IRD phenotype (macular dystrophy) constitutes approximately 30% of all IRD cases [13, 14]. Many macular dystrophies present with chorioretinal atrophy, with a similar appearance to GA in AMD [15]. In this study, we defined macular dystrophy as caused by nuclear or mitochondrial variants with disease attributable to a single gene (i.e. monogenic) or a pair of genes (i.e. digenic), noting that not all responsible genes have been identified thus far, as opposed to complex retinal disorders such as AMD, where the presence of any one of the disease-associated variants signifies an altered risk of disease but is not causative [16]. Voretigene neparvovec-rzyl, the first gene replacement therapy for biallelic mutations in *RPE65* has been approved globally [17, 18]. Trials of ocular gene therapy for at least 10 additional IRDs are underway [19, 20]. CRISPR-Cas9 gene editing technologies are also in development [21], and stem cell treatment clinical trials have been underway for both Stargardt macular dystrophy and atrophic AMD [22].

Distinguishing between atrophy due to AMD or that caused by macular dystrophies remains a clinical challenge, and the potential for misdiagnosis of AMD-mimicking macular dystrophies has been well recognized in the literature [15]. With different emerging treatments for both atrophic AMD and macular dystrophies, there are now greater implications for misdiagnoses. A UK study found that 5% of a cohort diagnosed with intermediate AMD or GA secondary to AMD harboured variants in genes potentially associated with autosomal dominant macular dystrophy [23], making their diagnosis more uncertain.

Genetic testing is helpful in diagnosing macular dystrophy, with a diagnostic rate of approximately 60% in establishing the causative gene [24], but is not commonly performed in real-world ophthalmology practice because of its availability and cost [25]. Furthermore, AMD cannot be confirmed by genetic testing, and only polygenic risk scores are available [26]. In the European Genetic Database (EUGENDA), several autosomal dominant macular dystrophies were identified in the AMD cohort [23]. However, the prevalence of potential misdiagnosis of macular dystrophies as AMD in a real-world clinical ophthalmology setting has not been investigated. Accurate diagnosis of GA is paramount to ensure that patients do not miss the opportunity for clinical trials and treatments when they become available, and also do not receive treatments targeted at AMD. Patients incorrectly enrolled in AMD clinical trials may confound clinical trial findings and cloud our understanding of potential treatment options and their efficacy. This is compounded by a paucity of chairside resources for eye care practitioners on distinguishing AMD from AMD-mimicking macular dystrophies to support referrals to targeted genetic testing.

The aims of this study are:

1. To determine the real-world frequency of the potential clinical misdiagnosis of macular dystrophies as atrophic AMD in a large Australian general ophthalmology practice.
2. To identify phenotype characteristics and clinical imaging modalities most useful for identifying clinical characteristics associated with inherited macular dystrophies.
3. To establish preliminary guidance for clinicians to improve the diagnosis of macular atrophy from AMD in practice, and thereby target cost-efficient genetic testing.

## Materials and methods

This study is a retrospective clinical record audit of electronic patient records from a large private general ophthalmology practice in Melbourne, Australia (Eye Surgery Associates [ESA]). To identify the shared phenotypic features between maculopathies, Table 1 shows common macular dystrophies with phenotypic characteristics similar to those of atrophic AMD.

**Table 1.** Common inherited macular dystrophies with similar phenotypic characteristics as atrophic age-related macular degeneration.

Phenotype	Associated genes	Inheritance pattern	Reported phenotypic characteristics
Central areolar choroidal dystrophy	*PRPH2*	AD	Central geographic atrophy and peripapillary atrophy [23] Speckled FAF pattern, possible reticular pseudodrusen [27]
	*CDHR1*	AR	Outer retinal/RPE atrophy, Macular chorioretinal Atrophy, or Bull’s-eye maculopathy [28] Yellowish flecks
Late-onset Stargardt disease	*ABCA4*	AR	Outer retinal/RPE atrophy Yellowish flecks in the retina, irregular and prominent on autofluorescence imaging [29]
Stargardt-like macular dystrophies	*ELOVL4* *PROM1*	AD	Central atrophy May have flecks or specific autofluorescence patterns [29]
Pattern dystrophy	*PRPH2*	AD	Irregular macular pigmentary changes or atrophy patterns [30] May have flecks or specific autofluorescence patterns
Adult-onset vitelliform macular dystrophies	*PRPH2* *BEST1* *IMPG1* *IMPG2*	AD	Central vitelliform lesions and marked autofluorescence [15]
Best vitelliform dystrophy	*BEST1*	AD	Central vitelliform lesions and marked autofluorescence [15] Macular atrophic changes in late stages
Mitochondrial macular dystrophies	m.3243A > G *ABCC6*	Mitochondrial maternal inheritance	Systemic symptoms, such as hearing loss and diabetes [31]
Late-onset retinal degeneration	*C1QTNF5*	AD	Well-demarcated macular atrophy Drusen-like yellow deposits [32]
Occult macular dystrophy	*RP1L1*	AD	Multifocal macular atrophy [23]
North Carolina macular dystrophy	*MCDR1* locus on chromosome 6 *MCDR3* on chromosome 5 *PRDM13*	AD	Macula atrophy and drusen-like deposits [33, 34]
X-linked juvenile retinoschisis	*RS1*	X-linked	Macular atrophic changes in late stages May have peripheral retinoschisis [35]

AD: autosomal dominant; AR: autosomal recessive; RPE: retinal pigmented epithelium.

### Case identification and sampling strategy

ESA has 19 ophthalmologists and offers all ophthalmic specialties, including tertiary-level medical retina and retinal electrophysiology services. The shared practice electronic database (Best Practice, Bundaberg, Australia, bpsoftware.net) includes approximately 180,000 individual patient files since its establishment in 1995, each of which records at least one patient visit (number of records obtained by database interrogation).

A database search (between 1 January 1995 and 5 June 2023) will be conducted to identify eligible patient records using the following AND/OR search terms: ‘geographic atrophy’, ‘GA’, ‘macular degeneration’, ‘AMD’, and ‘ARMD’. An estimated 4000 records and names of their participating ophthalmologists will be identified through keyword searches. The search will be limited to the records of 14 ophthalmologists who opted in for their patients to be included in the audit (14/19 ophthalmologists, approximately 74% records). Ophthalmologists who opted-in practice in Medical and Surgical Retina, Glaucoma, Cornea and Anterior Segment, Uveitis and Comprehensive Ophthalmology. Ophthalmologists who opted out practice in Medical and Surgical Retina, Cornea and Anterior Segment, Oculoplastics and Comprehensive Ophthalmology, similar to the included ophthalmologists.

All patients attending ESA had complete medical and ophthalmic history recorded and a complete eye examination, including visual acuity assessment (ETDRS chart), intraocular pressure measurement (Tonopen, Reichert, New York, USA; or iCare, Icare Finland), evaluation of pupils, slit lamp biomicroscopy, and dilated indirect ophthalmoscopy with a written description of the findings.

For patients with retinal disease, almost all (>95%) underwent optical coherence tomographic (OCT) scanning (Stratus [OCT3] and Cirrus, Zeiss, Oberkochen, Germany; Spectralis HRA + OCT, Heidelberg Engineering, Heidelberg, Germany) including scout *en-face* near infra-red (NIR) retinal images. Imaging was performed by a common group of technicians on shared equipment. In typical real-world clinical practice, compared to clinical trials, not all patients have complete multimodal imaging. Approximately 50% of patients had colour fundus photographs (CFP; Topcon TRC-50EX, Tokyo, Japan; 35°). A minority (<20%) underwent fundus autofluorescence (FAF, 488 nm) imaging (Spectralis HRA + OCT, Heidelberg Engineering, Heidelberg, Germany; author estimates).

### Participant record filtering

**Figure 1.**
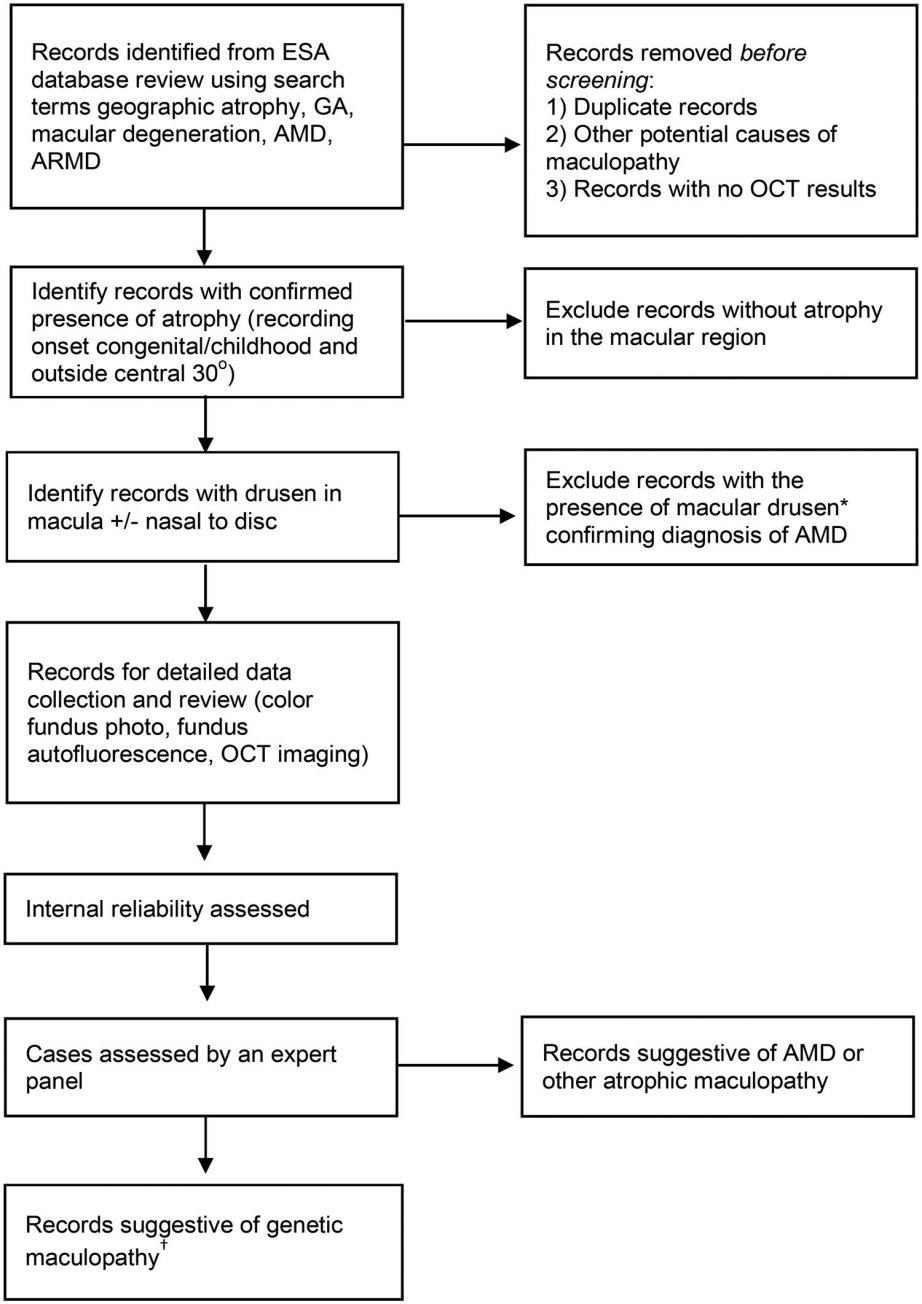
Study flow. *Drusen based on the Beckman Initiative for Macular Research Classification Committee standards and defined as extracellular deposits beneath the retinal pigmented epithelium >63 µm) [1]. ^†^Additional variates at this stage include the impact of ophthalmologist (retinal/general), imaging modality, year of most recent image.

Electronic patient records will be filtered in a stepwise manner to distinguish suspected macular dystrophy cases from those diagnosed with atrophic AMD (Figure 1). The records will be reviewed as follows:

1. Exclude duplicate records from the database search;
2. Eyes with confounding medical and ocular history potentially indicative of other causes of maculopathy, including oral steroid exposure, retinotoxic medications (e.g. hydroxychloroquine), ocular trauma, retinal vein occlusion, and other major retinal pathologies; macular laser (other than thermal laser or photodynamic therapy for choroidal neovascularization); myopia >6D, including prior to cataract surgery will be excluded (to exclude myopic maculopathy);
3. Exclude records without OCT scanning;
4. Confirm the presence of one or more foci of atrophy within 3000 µm of the centre of the fovea (AREDS grid) in one or both eyes using any imaging modality available. Rare cases of congenital/childhood onset of macular atrophy will be included as suspects for macular dystrophy, irrespective of drusen;
5. We will then review all cases (including historical data) and identify records with macular drusen (defined as extracellular deposits beneath the RPE >63 µm in diameter) based on all imaging from any available visits. Patients with macular drusen in one or both eyes at any time point will be diagnosed with AMD, based on CFP (using Beckman Initiative for Macular Research Classification Committee standards [1] and/or OCT scanning will be excluded as a suspected macular dystrophy case. At this data filtering stage, other forms of drusen, including drusen nasal to the optic disc will not be considered diagnostic of AMD and included as suspect macular dystrophy.

### Participant record review and data abstraction

The remaining records will be classified based on medical history and all clinical imaging from all visits to re-evaluate their maculopathy diagnoses in both eyes.

Data extraction will be performed using standardized data collection forms (Table 2). Data include demographic information (age and sex), age at symptom onset, and grading of retinal images from CFP, OCT scans, and NIR and FAF images where available.

**Table 2.** Proposed abstraction chart to identify people with macular dystrophy.

ESA ID	ID	
Demographics		
Age (years)		
Age of symptom onset (years)		
Sex	Male Female X	
Retina ophthalmologist	Yes No	
Date of exam (first exam)	Date of first exam	Date (last exam)/NA
Retinal imaging		
Eye	Right Left	
Refraction		
Eye included	Yes No	Yes No
Visual acuity		
FAF performed	Yes No	Yes No
FAF atrophy (select one)	Uni-lobular Multi-lobular	Uni-lobular Multi-lobular
FAF lesion (select any)	Speckled Trickling (if yes to either) Nasal location Temporal location	Speckled Trickling (if yes to either) Nasal location Temporal location
FAF-based diagnosis	AMD IRD Unsure	AMD IRD Unsure?
NIR images with OCT scan	Yes No	Yes No
(if yes) grading of atrophic lesions on NIR image	Uni-lobular Multi-lobular (if yes to either) Nasal location Temporal location	Uni-lobular Multi-lobular (if yes to either) Nasal location Temporal location
NIR-based diagnosis	AMD IRD Unsure	AMD IRD Unsure?
CFP performed	Yes No	Yes No
CFP features (select any)	Yellow flecking central Yellow flecking peripheral Pigment central Pigment peripheral	Yellow flecking central Yellow flecking peripheral Pigment central Pigment peripheral
CFP-based diagnosis	AMD IRD Unsure	AMD IRD Unsure?
OCT performed	Yes No	Yes No
OCT (select any)	Reticular pseudodrusen Flecks Lesion under fovea	Reticular pseudodrusen Flecks Lesion under fovea
OCT-based diagnosis	AMD IRD Unsure	AMD IRD Unsure?

CFP: colour fundus photography; FAF: fundus autofluorescence; NIR: near-infrared reflectance; OCT: optical coherence tomography. Participants at this stage have been screened for the presence of atrophy without drusen (according to Figure 1).

Retinal images will be graded using established clinical criteria:

1. From FAF images, macular lesions will be graded as uni- or multi-lobular [36], and for the presence of speckled [7] or trickling [8] atrophy. The nasal or temporal location relative to the fovea will also be recorded;
2. When FAF images are not available, these data will be identified from NIR *en-face* images accompanying the OCT line scans [36];
3. From the CFP photos, the presence of yellow flecks and pigment in the macula and/or periphery will be recorded;
4. OCT scans will be evaluated for the presence of flecks, and subfoveal lesions.

In addition, all CFP, NIR, and FAF images will each be classified as typical or atypical for AMD, first using single-modal, then multimodal modalities compared to the above reference standards. We consider typical AMD to be a bilateral condition of individuals 60 years or older, characterized by development of drusen at the macula, which typically undergoes a life-cycle culminating in GA. We will consider atypical features of AMD to include unilaterality, onset younger age, lack of drusen at any time point and unusual patterns of atrophy, for example, larger area that exceeds the central 30°, or speckled pattern on FAF.

Data extraction will be initially performed by a single reviewer (D.M.), who will be trained on data collection and retinal image grading using a panel of standard images similar to reference data for AMD [36–38]and IRD [39, 40], including distinguishing between drusen and flecks, and a reference panel of genetically confirmed IRD. Data accuracy will be monitored by a medical retinal ophthalmologist (H.M.) with >30 years of clinical experience in degenerative retinal diseases. Any discrepancies in the data abstraction or image grading will be reviewed jointly and discussed to resolve any issues.

The Cohen’s kappa index will be used to assess image grading reliability. One hundred cases will be assessed initially (estimated as 20% of the dataset), and 50 NIR and 50 FAF images will be randomly selected and reassessed by the same grader (as typical/atypical). We will consider an kappa >0.80 to be indicative of strong agreement, and 0.60–0.79 to indicate moderate agreement [41]. For kappa values between 0.40 and 0.60, images will be reviewed by the Principal Investigator. If the kappa value is below 0.40, training will be re-undertaken, and image grading reliability will be repeated.

### Case review and expert consensus diagnosis

The final diagnosis of screened patient files will be made by a panel of experts (including medical retina ophthalmologists and academic optometrists with expertise in AMD and IRDs), and consensus will be reached to classify the case as either suggestive of genetic maculopathy, or AMD, or other retinal conditions marked by atrophic maculopathy. Medical records, including pedigrees, genetic test results and retinal images from putative cases will be considered by the panel to support diagnosis. Given that our study is of records in a real-world clinical setting, the cases that we audit have not had molecular sequencing to confirm a diagnosis of macular dystrophy. To support diagnosis, reference to de-identified images from a genetically confirmed cohort will be used as a standard for comparison.

### Data management and privacy

Data will be collected and managed using Research Electronic Data Capture (REDCap) electronic data capture tools hosted at The University of Melbourne [42, 43]. REDCap is a secure web-based software platform that provides audit trails for tracking data capture, manipulation, and export procedures. Access to study data will be restricted to members of the study team. The storage and destruction of data will be compliant with the Australian Privacy Principles [4] and the Australian National Statement on Ethical Conduct in Human Research [45]. Intermittent random audits of data quality will be conducted by the Principal Investigator.

### Data analysis plan

De-identified data will be imported into an appropriate program for statistical analysis (e.g. R Statistical Software). Figure 1 will be completed showing the number of participants excluded at each stage based on the likely diagnosis of AMD or other diagnoses with macular atrophy.

Due to the clinical review design of this study, the sample size could not be calculated a priori. Based on previous database searching identifying about 4000 patients with AMD, and the published literature showing that approximately 1.4% of cases presenting with GA have pathogenic IRD variants, we estimate to identify up to 40 subjects (1% of returned records) with phenotypic characteristics associated with macular dystrophies [23].

Descriptive data will be presented as *n* (%) for the number and percentage of people suspected of having genetic maculopathy. Differences in proportions will be examined using the χ^2^ or Fisher’s exact test. Statistical significance will be set at *p* < .05. We will evaluate differences in outcome by year of last visit (before vs. after 2015, to reflect both contemporary ophthalmology practice and the year of a significant equipment upgrade at ESA), ophthalmologist seen (retinal specialist/general ophthalmologist), and FAF images available (yes/no) For factors significantly associated with the outcome, we will report the outcome for each group separately.

### Ethics

Human Research Ethics Committee (HREC) approval for a Low Risk and Negligible Risk project was sought and received by the Royal Australian and New Zealand College of Ophthalmologists HREC on 31 March 2023. All participants provided written informed consent to have their records audited for research purposes at the time of patient registration, with the patient registration and consent form periodically updated and re-signed since 1995. All methods and procedures will be performed in accordance with relevant guidelines and regulations.

### Participant information and counselling and follow-up studies

This study will generate a group of participants who have been diagnosed with AMD; however, a possible diagnosis of inherited macular dystrophy is suspected. Participating ophthalmologists will be notified of the possible diagnosis of macular dystrophy for their patients whose records were reviewed in the study and will have the option of notifying their patients. We recommend that participants be counselled that their diagnosed eye condition (in most cases AMD) has some atypical features and offered the option of participating in a follow-up evaluation of their eye disease, including genetic testing.

Further genetic investigations are outside the scope of retrospective auditing projects. However, participants may be able to undergo genetic screening to confirm their IRD diagnosis through clinical referrals or research [46]. We plan to report in subsequent studies (requiring additional patient consent) the number of cases in which genetic maculopathies are confirmed using prospective molecular testing.

## Discussion

This paper describes the challenge in differentiating between end-stage GA secondary to AMD and atrophy secondary to macular dystrophies in real-world, resource-limited, and image-limited ophthalmology practice. Genetic testing is currently recommended as a standard of care in Australia to confirm the genetic diagnosis of macular dystrophy [47], but it is performed in the minority in clinical practice due to resource constraints [25]. There is a clear role for clinical guidelines that can be used in the consulting suite to assist with the identification of patients who present with features of IRD and ensure that they are referred for further investigation and targeted genetic testing.

With progress in available treatments for both AMD and IRD, this study will help to highlight the clinical differences between both diseases to allow for more nuanced assessment and greater accuracy in diagnosis in routine clinical practice. This is particularly important as misdiagnosis represents the potential for contamination in patient populations for clinical trials, which may confound the results. Additionally, as therapies become available to prescribe, on an individual patient basis, accurate diagnosis is required so that the misattribution of clinical signs to other possible causes does not represent a barrier to accessing the most efficacious treatment for their IRD or result in the wrong treatment (e.g. treatments for GA).

Strength of the current study is the large dataset collected in the real-world setting with different ophthalmologists, which increase the external validation of the results, despite being from a single study setting. A key study limitation is that the diagnosis of macular dystrophy cannot be confirmed by genetic sequencing, which is outside the scope of the retrospective clinical audit. Molecular diagnosis is the gold standard for diagnosing IRD. In the present study, genetic confirmation is not possible if molecular testing results were not already a part of the medical records. To mitigate misclassification bias, we will include reference to a genetically confirmed cohort with atrophy from macular dystrophies as a standard for comparison both during image grading and expert consensus. In addition, we plan to report the number of cases in which genetic maculopathies are confirmed using prospective molecular testing. By reporting on this data, we hope to highlight steps towards best practice in screening and identifying patients with macular diseases suitable for targeted genetic testing.

Being a retrospective study of a real-world clinical setting, data relies on information available in clinical records and results are prone to information bias from completeness of imaging and records. To assess the impact of information availability on the outcome, we will examine differences in misdiagnosis rates between year of examination, ophthalmologist seen, and availability of FAF images If these factors are associated with the outcome, we will report the outcome for each group separately to improve the interpretation of the data and ensure that results are relevant to contemporary practice.

Based on local real-world data, this study aims to establish preliminary guidance for clinicians to recognize the distinction between macular atrophy from AMD in their practice, thereby understanding which patients would benefit from IRD genetic sequencing. Future work includes establishing consensus by international experts to create more generalized guidelines that facilitate distinction between the macular diseases, representing the spectrum of macular diseases seen globally. In publishing this study protocol, we hope to promote transparency and support future studies aiming to undertake a similar process to evaluate macular disease misdiagnosis in real-world practice.

In summary, the findings of this study can be used to create clinical resources that can provide greater acuity to clinicians to avoid misdiagnosis and allow all patients access to optimal care. Improving the diagnostic accuracy of atrophy secondary to either AMD or inherited macular dystrophies, while ensuring cost-effective genetic testing, will improve the targeted treatment of macular diseases when new treatments become available.

## Data Availability

De-identified data will be available from the principal investigator (H.G.M.) upon reasonable request.
